# Supplementary figures and images for: Costs, effectiveness, and safety associated with Chimeric Antigen Receptor (CAR) T-cell therapy: Results from a comprehensive cancer center
Source: PLoS One. 2022 Dec 9;17(12):e0278950. doi: 10.1371/journal.pone.0278950 (PMC9733886; doi:10.1371/journal.pone.0278950)

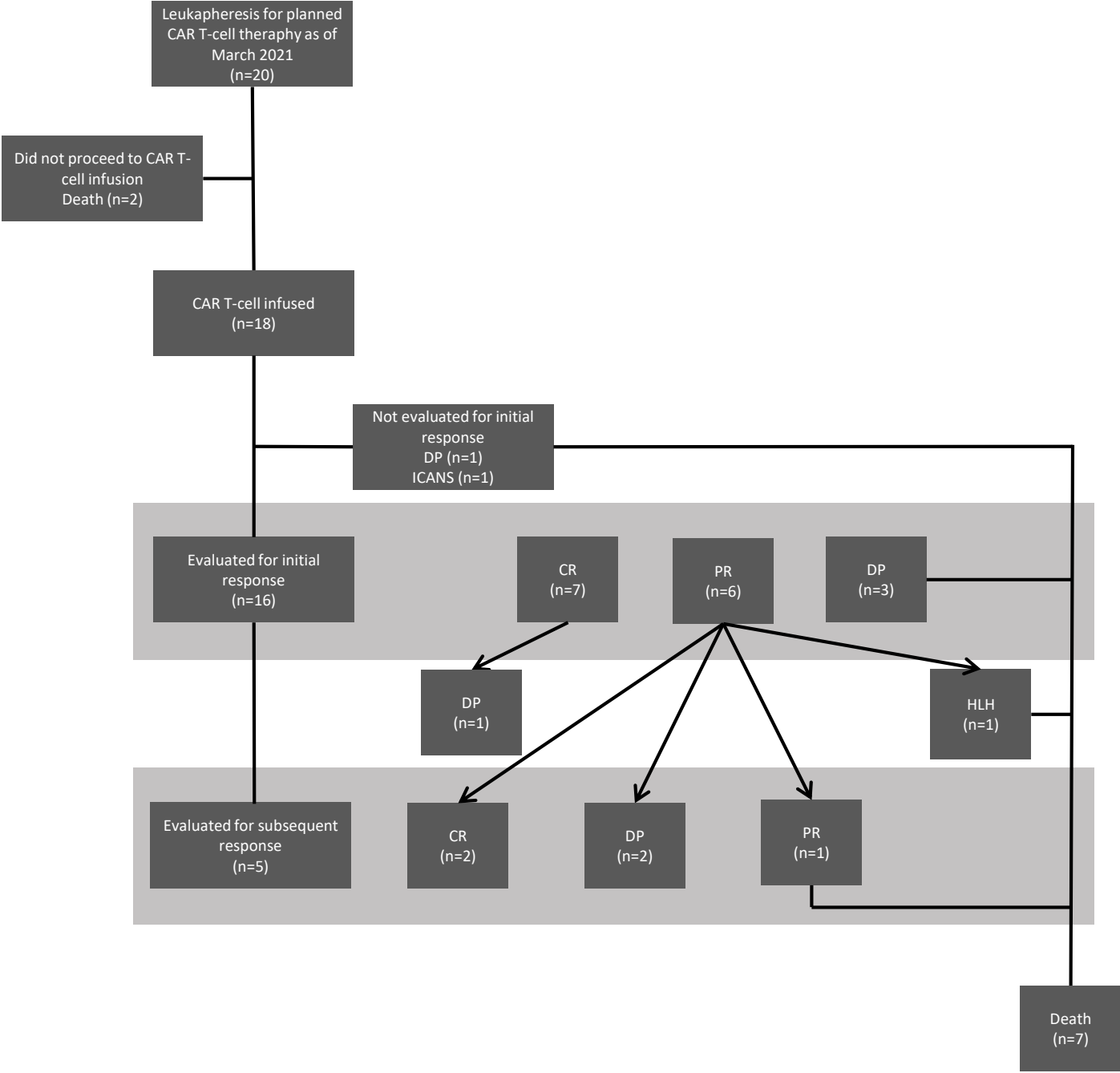

Supplement: S1 Fig — CAR, chimeric antigen receptor; DP, disease progression; ICANS, immune effector cell-associated neurotoxicity syndrome; CR, complete response; PR, partial response; HLH, Hemophagocytic lymphohistiocytosis. (PDF) [file pone.0278950.s001.pdf]
