# Supplementary material for: Costs, effectiveness, and safety associated with Chimeric Antigen Receptor (CAR) T-cell therapy: Results from a comprehensive cancer center
Source: PLoS One. 2022 Dec 9;17(12):e0278950. doi: 10.1371/journal.pone.0278950 (PMC9733886; doi:10.1371/journal.pone.0278950)
Supplement: Attachment — Submitted filename: Response to Reviewers.docx [file pone.0278950.s002.docx]

**RESPONSES TO THE COMMENTS FROM THE EDITORS AND REVIEWERS**

We acknowledge the important issues that were raised by Editors and Reviewers, and we believe that the changes made to the manuscript resulted in substantial improvement of the overall quality and clarity of the work. Comments are individually addressed below.

## Journal Requirements

**1. Please ensure that your manuscript meets PLOS ONE's style requirements, including those for file naming.**

R: We have changed the manuscript layout and files naming to meet the PLOS ONE style requirements.

**2.** **We note that you have indicated that data from this study are available upon request. PLOS only allows data to be available upon request if there are legal or ethical restrictions on sharing data publicly. For more information on unacceptable data access restrictions, please see http://journals.plos.org/plosone/s/data-availability#loc-unacceptable-data-access-restrictions.**

**In your revised cover letter, please address the following prompts:**

**a) If there are ethical or legal restrictions on sharing a de-identified data set, please explain them in detail (e.g., data contain potentially sensitive information, data are owned by a third-party organization, etc.) and who has imposed them (e.g., an ethics committee). Please also provide contact information for a data access committee, ethics committee, or other institutional body to which data requests may be sent.**

**b) If there are no restrictions, please upload the minimal anonymized data set necessary to replicate your study findings as either Supporting Information files or to a stable, public repository and provide us with the relevant URLs, DOIs, or accession numbers. For a list of acceptable repositories, please see http://journals.plos.org/plosone/s/data-availability#loc-recommended-repositories.**

**We will update your Data Availability statement on your behalf to reflect the information you provide.**

R: In the revised cover letter we transcribed the justification written by the Data Protection Officer from the Portuguese Oncology Institute of Porto (IPO-Porto) of the ethical and legal restrictions on sharing a de-identified data set. IPO-Porto Data Protection Officer provided the following explanation: *“This study is based on a very small sample (n=20), so we understand that the particularity of this study, given the current state of the art, may lead to an increased risk of violating the privacy of our patients through a possible identification of the data subject. However, this concern is the result of the ethical and legal obligations to which our institution is bound in light of the current guidelines and recommendations and legislation”*. We also updated the manuscript with institutional contacts information to which data requests may be sent as follows: Data requests may be sent to the Data Protection Officer ([epd@ipoporto.min-saude.pt](mailto:epd@ipoporto.min-saude.pt)) and to the Department of Onco-hematology at IPO-Porto (s.oncohematologia@ipoporto.min-saude.pt).

**3. Please review your reference list to ensure that it is complete and correct. If you have cited papers that have been retracted, please include the rationale for doing so in the manuscript text, or remove these references and replace them with relevant current references. Any changes to the reference list should be mentioned in the rebuttal letter that accompanies your revised manuscript. If you need to cite a retracted article, indicate the article’s retracted status in the References list and also include a citation and full reference for the retraction notice.**

R: We updated the reference list with three relevant recent publications and replaced the reference from an abstract presented in a conference with the recently published manuscript (Kuhnl et al 2022) as follows:

Gajra A, Zalenski A, Sannareddy A, Jeune-Smith Y, Kapinos K, Kansagra A. Barriers to Chimeric Antigen Receptor T-Cell (CAR-T) Therapies in Clinical Practice. Pharmaceut Med. 2022;36(3):163-71. Epub 20220607. doi: 10.1007/s40290-022-00428-w. PubMed PMID: 35672571; PubMed Central PMCID: PMC9217916.

Maziarz RT, Yang H, Liu Q, Wang T, Zhao J, Lim S, et al. Real-world healthcare resource utilization and costs associated with tisagenlecleucel and axicabtagene ciloleucel among patients with diffuse large B-cell lymphoma: an analysis of hospital data in the United States. Leuk Lymphoma. 2022;63(9):2052-62. Epub 20220414. doi: 10.1080/10428194.2022.2060503. PubMed PMID: 35422192.

Valade S, Darmon M, Zafrani L, Mariotte E, Lemiale V, Bredin S, et al. The use of ICU resources in CAR-T cell recipients: a hospital-wide study. Ann Intensive Care. 2022;12(1):75. Epub 20220817. doi: 10.1186/s13613-022-01036-2. PubMed PMID: 35976532; PubMed Central PMCID: PMC9385897.

Kuhnl A, Roddie C, Kirkwood AA, Tholouli E, Menne T, Patel A, et al. A national service for delivering CD19 CAR-Tin large B-cell lymphoma - The UK real-world experience. Br J Haematol. 2022;198(3):492-502. Epub 20220429. doi: 10.1111/bjh.18209. PubMed PMID: 35485402.

## Reviewers' comments

### **Reviewer #1:**

We thank the reviewer for his careful reading of the manuscript and their constructive remarques.

**[…] very few corrections need to be made namely, a few abbreviations need to be highlighted PMBL, lin2 162, NT, line 182.**

R: Errors in abbreviations have been corrected accordingly.

### **Reviewer #2:**

**1. Response rate: the authors may choose whether the denominator is 20 (all patients included) or 18 (all patients treated), but never 16 (patients evaluated). Imagine now that I have a trial and 10 patients are treated: 8 patients are never evaluated for response because they die early or experience disease progression and 2 patients are evaluated. Both patients respond to therapy. You would never say that the response rate is 100%, right?**

R: We thank the reviewer for pointing this out. We have now followed the appropriate suggestion from two of the reviewers and updated the effectiveness analysis to include the 20 participants in the denominator, consistent with the definition an intention-to-treat analysis.

**2. The sentence starting on line 188: "Four hospitalizations..." is unclear.**

R: We have rewritten the sentence in a different way to make it clearer.

**3. Line 227: "progressed/diseased" or "progressed/deceased"?**

R: The word diseased was corrected by deceased.

**4. I would prefer the term "ICANS" to "NT".**

R: We agreed that that NT is not the accurate term to describe these events. We replaced it with ICANS throughout the manuscript.

**5. Please define high-dose and low-dose corticosteroids. By the way, is this distinction important?**

R: We agree with the reviewer's comment, and we have added information about the definition for high-dose and low-dose corticosteroid use to the manuscript. High dose is defined as 1000mg of intravenous methylprednisolone as a single day dose or 1mg/kg every 12 hours and low dose corticosteroids is defined as dexamethasone up to 10mg every 6 hours or equivalent dose.

In our point of view this distinction is relevant because the high dose of methylprednisolone 1000 mg/day used for grade 4 CRS/ICANS treatment, that is according to the current recommendations (Santomasso, B D, et al. Management of Immune-Related Adverse Events in Patients Treated With Chimeric Antigen Receptor T-Cell Therapy: ASCO Guideline, Journal of Clinical Oncology 2021 39:35, 3978-3992), is substantially higher than the corticosteroid dose recommended for the other situations.

### **Reviewer #3:**

**1. As this is an intention-to-treat analysis, I would prefer to add the 4 patients excluded (two died due to disease progression before CAR-T, two died after CAR T due to toxicity or progression.**

R: We thank the reviewer for pointing this out. We have now followed the appropriate suggestion from two of the reviewers and updated the effectiveness analysis to include the 20 participants in the denominator, consistent with the definition an intention-to-treat analysis.

**2. Cell of origin is given for twelve patients with 8 missing. I think it is better to have two missing as COO seldom is given for PMBCL or transformed lymphomas (3 + 3 patients).**

R: We agree with the reviewer in this remark. We have changed table 1 to present the distribution of cell of origin exclusively for DLBCL patients.

**3. Bridging therapy is given, but I could not find** **lymphodepletion (I assume FC or Bendamustin or neither)**

R: We agree that the description of lymphodepletion chemotherapy is of utmost relevance to characterize costs associated to CAR T-cell therapies and that information was included in table 1 notes as follows. After scheduling the infusion day (day 0) lymphodepletion chemotherapy is administered in the following way:

For Axicabtagene ciloleucel:

- intravenous ondansetron (8 mg) every 8 hours, at days -5, -4 and -3

- intravenous saline solution (2000 ml) per day, at days -5, -4 and -3

- intravenous cyclophosphamide (500 mg/m^2^) infused over 60 minutes, at days -5, -4 and -3

- intravenous fludarabine (30 mg/m^2^, maximum dose of 50 mg) infused over 30 minutes, at days -5, -4 and -3

For Tisagenlecleucel:

- intravenous ondansetron (8 mg) every 8 hours, at days -5, -4 and -3

- intravenous saline solution (2000 ml) per day, at days -5, -4 and -3

- intravenous cyclophosphamide (250 mg/m^2^) infused over 60 minutes, at days -5, -4 and -3

- intravenous fludarabine (25 mg/m^2^, maximum dose of 50 mg) infused over 30 minutes, at days -5, -4 and -3

**4. Minor language deficiencies can be fixed during editorial review.**

R: Inaccuracies in language and spelling capture by the reviewers were corrected Additional comments and suggestions are welcome and appreciated.
